# Keratinocyte Migration in the Developing Eyelid Requires LIMK2

**DOI:** 10.1371/journal.pone.0047168

**Published:** 2012-10-05

**Authors:** Dennis S. Rice, Gwenn M. Hansen, Feng Liu, Mike J. Crist, Matthew M. Newhouse, David Potter, Nianhua Xu, Alejandro Abuin, Peter J. Vogel, Brian P. Zambrowicz

**Affiliations:** Lexicon Pharmaceuticals Incorporated, The Woodlands, Texas, United States of America; University of Birmingham, United Kingdom

## Abstract

*In vitro* studies have identified LIMK2 as a key downstream effector of Rho GTPase-induced changes in cytoskeletal organization. LIMK2 is phosphorylated and activated by Rho associated coiled-coil kinases (ROCKs) in response to a variety of growth factors. The biochemical targets of LIMK2 belong to a family of actin binding proteins that are potent modulators of actin assembly and disassembly. Although numerous studies have suggested that LIMK2 regulates cell morphology and motility, evidence supportive of these functions *in vivo* has remained elusive. In this study, a knockout mouse was created that abolished LIMK2 biochemical activity resulting in a profound inhibition of epithelial sheet migration during eyelid development. In the absence of LIMK2, nascent eyelid keratinocytes differentiate and acquire a pre-migratory phenotype but the leading cells fail to nucleate filamentous actin and remain immobile causing an eyes open at birth (EOB) phenotype. The failed nucleation of actin was associated with significant reductions in phosphorylated cofilin, a major LIMK2 biochemical substrate and potent modulator of actin dynamics. These results demonstrate that LIMK2 activity is required for keratinocyte migration in the developing eyelid.

## Introduction

Regulation and remodeling of the actin cytoskeleton are critical events affecting cell-cell and cell-extracellular matrix interactions during tissue morphogenesis and wound repair [1], [2]. Molecular signaling pathways that control actin dynamics are highly conserved among species and similar phenotypes are often observed in genetic models [1]. Closure of the eyelid in mammals occurs during embryogenesis. In mice, eyelid closure initiates on the tips of the eyelid folds at embryonic day 15 (E15) and is completed approximately 24 hours later, on E16. Fusion of opposing eyelids occurs through extension of the eyelid fold in the form of a sheet of migrating keratinocytes and surrounding periderm cells. Fusion begins in the temporal and nasal canthi and progresses towards the center of the eye [3]–[5].

A surprising number of molecules in diverse signaling pathways are involved in eyelid closure. For example, genetic knockout of either epidermal growth factor receptor (EGFR) or several of its ligands, such as EGF, transforming growth factor alpha (TGFα) and heparin binding-EGF-like growth factor (HB-EGF) leads to a developmental defect known as “eyes open at birth,” designated as EOB [6]–[10]. At the cellular level, the EOB phenotype is commonly associated with abnormally low accumulations of filamentous actin (F-actin) in the advancing eyelid epithelial sheet. Ras homolog gene family, member A (RhoA) is a GTPase protein known to induce accumulation of F-actin and focal adhesion complexes [11]. Rho-associated coiled-coiled kinase 1 and 2, known as ROCK1 and ROCK2, are important downstream effectors of RhoA and knockout of either ROCK1 or ROCK2 results in an EOB phenotype [12], [13]. Moreover, EGF is unable to stimulate phosphorylation of myosin light chain (MLC) in primary keratinocytes isolated from ROCK1 knockouts suggesting an impairment in assembly of actomyosin bundles which would normally contract and provide the mechanical force for epithelial sheet closure [12]. In addition to MLC, ROCKs are known to directly act on several additional biochemical substrates that affect actin filament assembly and cellular contractility [14].

Two of these targets are related proteins known as LIM motif-containing protein kinase 1 and 2 (LIMK1 and LIMK2) [15]–[17]. LIMKs have been implicated in controlling cell morphology, proliferation, neuronal differentiation, endocytosis and oncogenesis primarily via regulation of actin binding proteins (ABPs) such as cofilin 1, cofilin 2, and actin depolymerizing factor [16]–[19]. LIMKs directly regulate cofilin-induced actin severing by phosphorylation of Ser-3 in cofilin [20]–[23]. This post-translational modification abrogates cofilin binding to actin and promotes F-actin accumulation, lamellipodia and filopodia formation and maturation of focal adhesion complexes [20], [22]–[24]. Recently, LIMKs were proposed as potential targets for cancer cell metastasis because suppressing their expression or diminishing their biochemical function, reduced three-dimensional collective cell invasion *in vitro* [25]. In the present study we show for the first time that LIMK2 is critical for cell migration in an *in vivo* context. Specifically, in the developing mouse eyelid, a system of both cellular and genetic relevance to tumor invasion, LIMK2 is required for the assumption of a migratory phenotype by epithelial keratinocytes.

**Figure 1:**
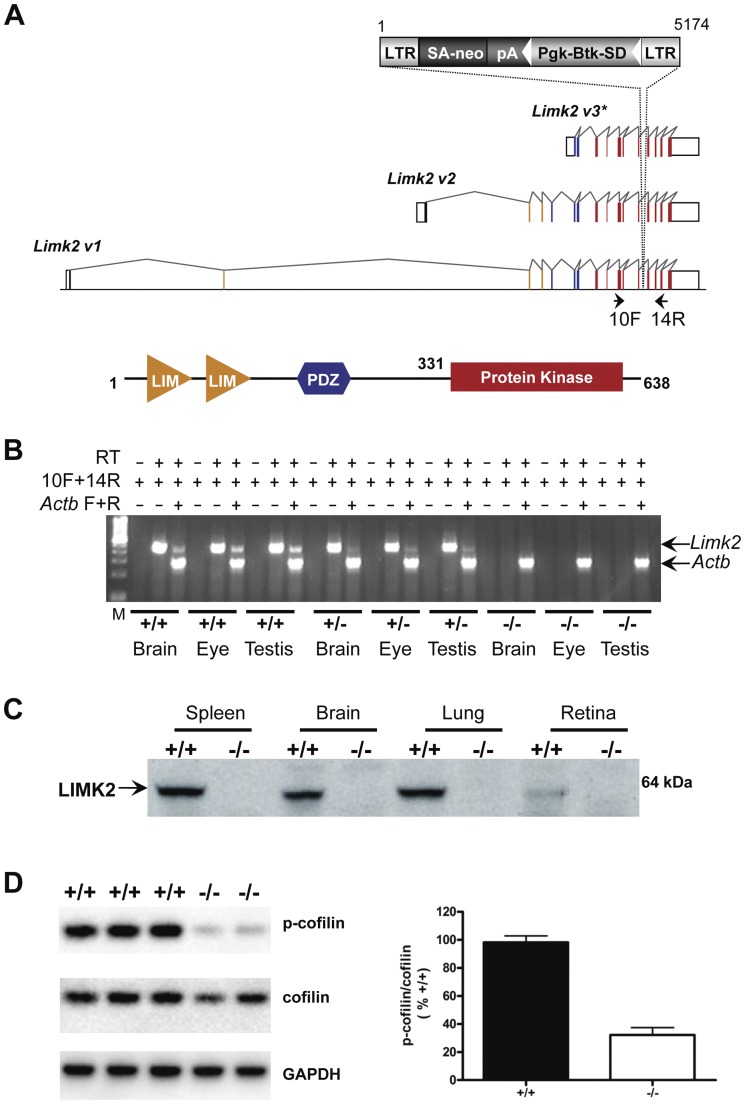
Generation and characterization of *Limk2-*mutant mice. (A) Retroviral gene trap vector VICTR48 (EU676804) was used to produce OmniBank clone OST80053, which contains an insertion within intron 12 of the *Limk2* gene. The *Limk2* transcript variants 1–3 (v1–3) represent protein isoforms a-c, respectively, according to current NCBI Reference Sequence annotation for accessions NM_010718.3, NM_173053.1, and NM_001034030.1 (intron/exon numbering based on transcript v1). This mutation would be expected to truncate the *Limk2* gene product within the kinase domain following coding exon 12, disrupting all reported transcript variants of the *Limk2* gene. Open boxes denote untranslated exons, filled boxed denote coding exons. Exons that code for the LIM domains are shown in orange, those that code for the PDZ domain are shown in blue, and those that code for the kinase domain are shown in red. LTR, viral long terminal repeat; SA, splice acceptor sequence; *neo*, neomycin phosphotransferase gene; pA, polyadenylation sequence; *Pgk*, phosphoglycerate kinase-1 promoter; *Btk*-SD, Bruton's tyrosine kinase splice donor sequence. *The Limk2 v3 transcript is elsewhere referred to as Limk2t [45] or tLimk2 [46]. (B) RT-PCR expression analysis of *Limk2* transcripts. Endogenous *Limk2* transcription was detected in the brain, eye and testis of wild type (+/+) and heterozygous (+/−) mice. No endogenous *Limk2* expression was detected in homozygous (−/−) tissues. Primers 10F and 14R are complementary to *Limk2* exons 10 and 14/15, respectively, and amplify a product of 489 nucleotides. (C) Immunoblotting analysis with a rat monoclonal antibody that recognizes mouse LIMK2 residues 145–260, which includes the PDZ domain, revealed LIMK2 in several tissues from wild type mice (+/+) such as spleen, brain, lung and retina. LIMK2 was undetectable in these same tissues isolated from homozygous *Limk2* (−/−) mutant mice. (D) Mouse primary keratinocytes were cultured from neonatal animals and probed with antibodies that recognize phospho-cofilin, total cofilin or GAPDH. Cell cultures were obtained from several litters to quantify the ratio of p-cofilin to cofilin. There was a statistically significant reduction in p-cofilin levels in the knockout compared to wild type. The difference between the means was 66%±7 (*n* = 4 per genotype; P<0.0001; student's T test).

## Materials and Methods

### Generation of mutant ES cells and genotyping

A knockout allele of the *Limk2* gene was generated by gene trapping in 129S5SvEvBrd-derived embryonic stem (ES) cells as previously described [26], [27]. The precise location of gene trap vector insertion was determined by inverse genomic PCR using oligonucleotide primers complementary to the vector. Mutant mice were generated by microinjection of ES cells into C57BL/6-Tyr*^c-Brd^* (albino) blastocysts using standard methods [28]. The resulting chimeras were bred to C57BL/6-Tyr*^c-Brd^* (albino) females. Genotypes of the resulting F1 offspring (a pure hybrid genetic background containing 129S5SvEvBrd and C57BL/6-Tyr*^c-Brd^* chromosomes) were determined by quantitative real-time PCR as previously described [29]. In brief, DNA isolated from tail biopsy samples was assayed by real-time PCR for the *neo* gene, which is present in gene trap vector VICTR48 (EU676804). Genotypes were subsequently confirmed with a mutation-specific PCR assay using oligonucleotide primers (LTR-2: 5′- AAATGGCGTTACTTAAGCTAGCTTGC-3′, and OST80053 Rev: 5′- TAGGATTTCTTGTCTATCTTCAGCC-3′) which produce a mutant product of 238 bps.

**Figure 2:**
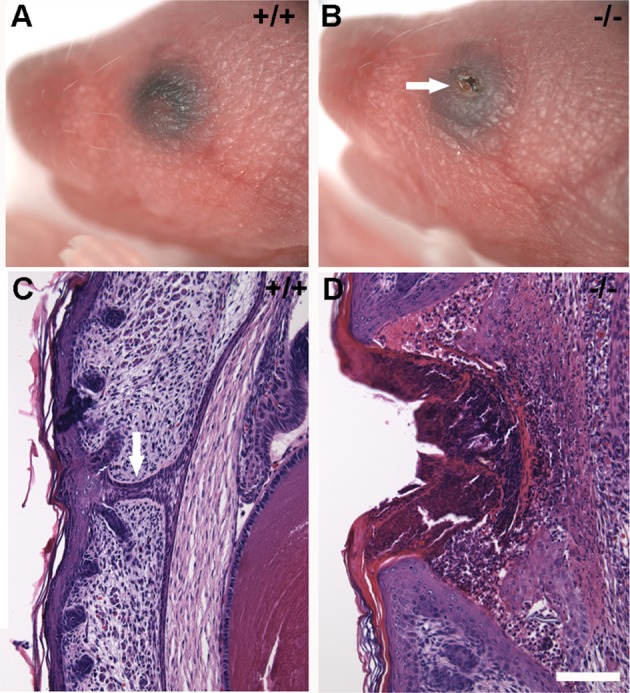
Ocular anatomy of newborn mice. (A) Normal appearance of newborn, PD 1.5 mice. Eyelids are fused and completely cover the ocular surface at this age in wild type mice (+/+). (B) Mice lacking *Limk2* exhibit an eyelid open at birth (EOB) phenotype. Upper and lower eyelids are separated and the ocular surface (arrow) is exposed. Hematoxylin and Eosin (H & E) stained paraffin sections of formalin-fixed specimens obtained at PD 1.5. (C) Wild type mice exhibit a zone of fusion between the upper and lower eyelids (arrow) that involves only the epithelium and not the basal layer of the epidermis. (D) The upper and lower eyelids are not fused in *Limk2* deficient mice. Numerous cellular infiltrates can be observed in this section. Scale bar in D = 100 μm.

### RT-PCR analysis

RNA was extracted from mouse tissues or cultured cells using a bead homogenizer and/or TRIzol reagent (Invitrogen, Carlsbad, CA) according to the manufacturer's instructions. Unless otherwise noted, reverse transcription (RT) was performed with SuperScript II (Invitrogen) and random hexamer primers according to manufacturer's suggestions (Life Technologies, Carlsbad, CA).

**Figure 3:**
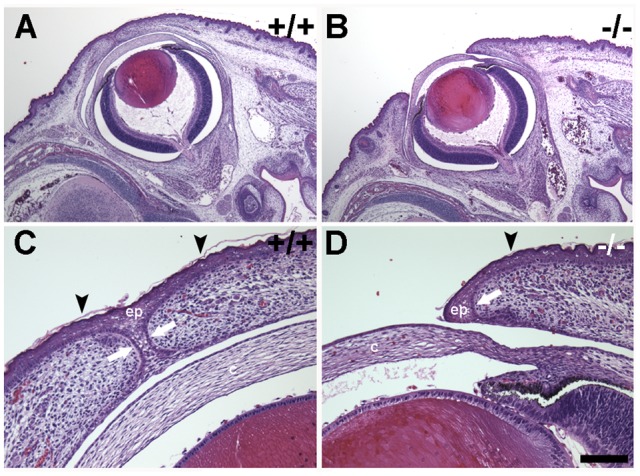
Ocular anatomy following eyelid fusion but prior to birth. H & E-stained paraffin sections of Bouin's-fixed specimens obtained at E18.5. (A) Eyelids are fused in wild type embryos (+/+) prior to birth. (B) Eyelids are not fused in *Limk2*-deficient embryos. With the exception of the open eyelids, the ocular histology is comparable to wild type. (C) Higher magnification view of a wild type eyelid opposition shows fusion of the eyelid epithelium (ep) with the initial stages of keratinization (arrowheads) in the superficial epithelium. The basal layer of epithelium is not involved in fusion (arrows). (D) The epithelium (ep) is apparent above the basal layer (arrow) of the epidermis. However, the cornea (c) is exposed prior to birth in the *Limk2*-deficient mice. Keratinization (arrowhead) is observed on the palpebral epidermis. Scale bar in A and B  = 500 μm and 100 μm in C and D.

To confirm the mutagenicity of the gene trap *in vivo*, RT-PCR amplification was performed on RNA isolated from wild-type, heterozygous and homozygous animal tissues. Reactions (35 cycles of 95°C, 30 seconds, 59°C, 45 seconds, 70°C, 60 seconds) were performed using primers complementary to *Limk2* exons flanking the insertion site (10F: 5′- TGATGCGGAGCCTGGACCACCCTAAT -3′ and 14R: 5′-GCCCAATGATCTCACA GAGAACGATCCC-3′). Oligonucleotide primers complementary to the mouse beta-actin gene (Actb, accession NM_007393) 5′-GGCTGGCCGGGACCTGACGGACTACCTCAT-3′ and 5′- GCCTAGAAGCACTTGCGGTGCACGATGGAG-3′ were used as an internal amplification control. All *Limk2* RT-PCR products were gel purified and verified by direct sequencing.

**Figure 4:**
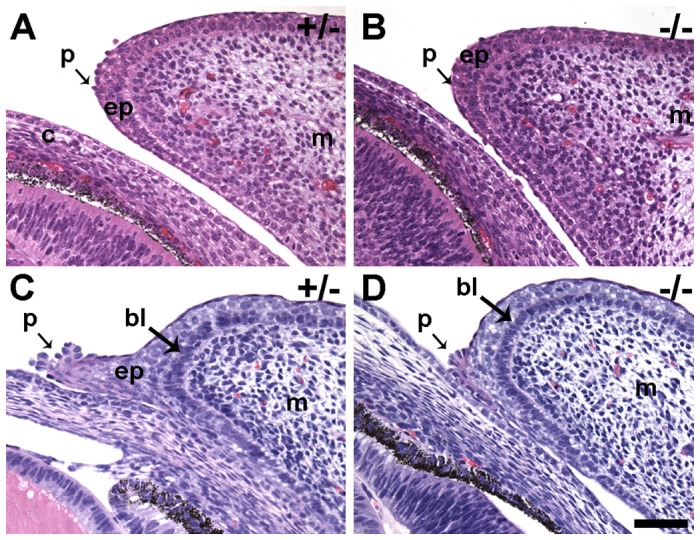
Ocular anatomy prior to eyelid fusion. H & E-stained paraffin sections of eyelids obtained from E14.5 (A and B) and E15.5 (C and D) embryos. No phenotypic differences are apparent in *Limk2* knockout (B) mice compared with control (A). The eyelid of both heterozygous (+/−) and homozygous (−/−) is largely comprised of mesenchyme (m) covered by epithelium (ep). The morphology of periderm (p) cells in the eyelid tip is round in contrast to the flat on the surface of the eyelid. Arrows in A and B point to specialized periderm cells that have changed from a squamous to a rounded morphology in preparation for epithelial sheet migration. A phenotype is clearly observed in E15.5 specimens. (C) The epithelium (ep) in heterozygous mice (+/−) has matured into a sheet of cells behind the rounded peridermal cells (p) that lead the migrating front across the cornea. The morphology of epithelial cells in the basal layer (bl) is clearly distinct from the adjacent mesenchymal cells (m) and the superficial epithelial cells. (D) Although rounded peridermal (p) cells are present in the *Limk2*-deficient eyelids, there is no evidence of epithelial sheet extension across the cornea. Scale bar  = 50 μm.

### Ethics Statement

Animals were handled in accordance with the ARVO Statement for the Use of Animals in Ophthalmic and Vision Research, and protocols were approved by the IACUC at Lexicon Pharmaceuticals.

**Figure 5:**
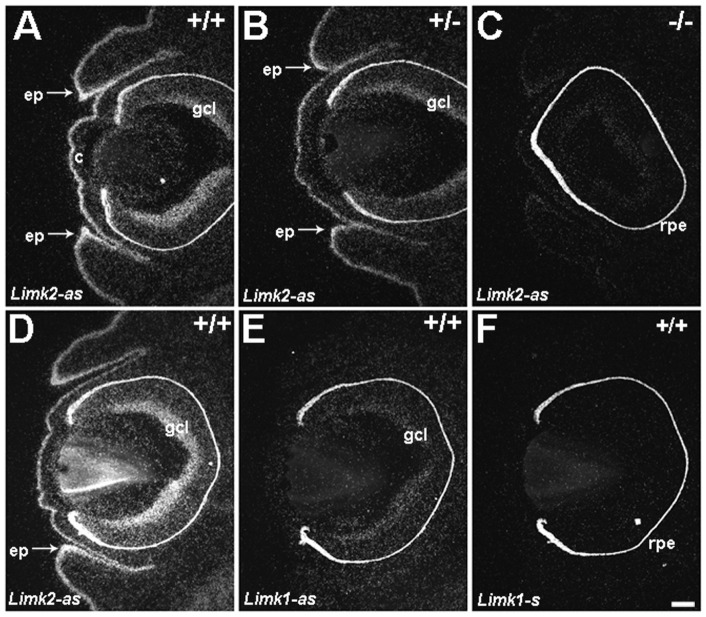
Expression of *Limks* in ocular tissues during eyelid closure. *In situ* hybridization in E15.5 specimens with radioactive riboprobes (as = antisense; s  =  sense) specific for either *Limk2* or *Limk1*. (A) High levels of *Limk2* are detected in the epithelium (ep) in both upper and lower eyelids in wild type mice (+/+). The epithelium in the cornea (c) and differentiated cells in the ganglion cell layer (gcl) of the retina express *Limk2*. The emerging epithelial sheets at the eyelid tips (arrows) exhibit more intense *Limk2* hybridization signals compared to adjacent epithelium. (B) Although the intensity of hybridization is decreased in the heterozygous (+/−) specimen, the distribution of *Limk2* is comparable to that in wild type. Higher levels of *Limk2* are present in the emerging epithelial sheets (arrows). (C) Homozygous mice, mounted and processed on the same slide as the specimens in A and B, show extremely low levels of hybridization. Three adjacent sections of another wild type E15.5 specimen hybridized with either the antisense *Limk2* (D), antisense *Limk1* (E) or sense *Limk1* (F). *Limk2* is expressed in the epithelium and gcl, whereas *Limk1* is detectable in the gcl. The intense signal in the retinal pigment epithelium (rpe in C and F) is an artifact of melanin that appears during dark field illumination and is present in all samples shown. Scale bar  = 100 μm.

### Histopathology, *in situ* hybridization and immunohistochemistry

Embryos were harvested from timed-matings between heterozygous mice. Plugs were recorded daily between 0700 and 0900. The day of plug observation is considered as embryonic day 0.5 (E0.5). The day of birth was considered postnatal day 0.5 (PD 0.5). Histopathology was conducted on tissue immersion fixed in either 10% neutral buffered formalin (Statlab, Lewisville, TX) or Bouin's fixative (LabChem, Pittsburg, PA). Tissues were processed in paraffin, cut at a thickness of 5 μm and stained with Hematoxylin and Eosin. Coverslips were applied with Permount (Electron Microscopy Sciences, Hatfield, PA). Digital images were acquired with a Hamamatsu ORCA II cooled CCD camera mounted on an Olympus BX60 microscope.

**Figure 6:**
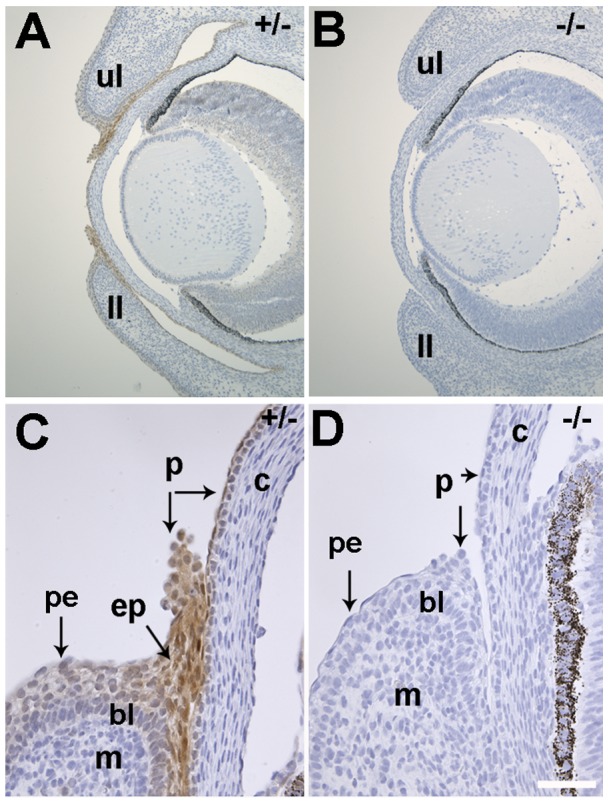
Distribution of LIMK2 in E15.5 ocular adnexa. (A) Low magnification view of cross sections through the ocular tissue (nuclei appear blue with Hematoxylin stain) in a heterozygous mouse (+/−) immuno-labeled with an anti-Limk2 monoclonal rabbit antibody (brown stain). High levels of LIMK2 are detected in both upper (ul) and lower (ll) eyelids. (B) LIMK2 is not detectable in homozygous (−/−) *Limk2*-deficient mice. (C) Higher magnification of the upper eyelid (ul) shown in A. Periderm cells (p) in both the epithelial sheet (ep) and cornea (c) express LIMK2. The migrating keratinocytes in the ep and the corneal epithelium contain LIMK2, whereas mesenchyme (m) is not labeled. Note the more intense brown LIMK2-specific signal in the epithelium of the eyelid sheet compared to adjacent palpebral epithelium (pe) or basal layer (bl) of the epidermis. (D) LIMK2 immunoreactivity was not observed in the knockout (−/−) tissue. Scale bar in A and B  = 200 μm and 50 μm in C and D.

The method of *in situ* hybridization analysis was performed as described in [30] on 16 µm-thick cryosections. Specimens were immersion-fixed in ice cold 3.2% paraformaldehyde (generated from 16% EM grade stock; Electron Microscopy Sciences, Hatfield, PA) in 0.1 M phosphate buffer containing 0.15 M NaCl (PBS; pH 7.3). Following overnight fixation, tissues were transferred to 20% sucrose in 0.1 M PBS overnight and then embedded in Tissue Freezing Medium (Triangle Biomedical Sciences, Durham, NC). A riboprobe was generated from a 521 bp, *Limk2*-specific cDNA fragment using PCR primers (F: 5′- CTACAATTAACCCTCACTAAAGGACCACCCTAAT-3′ and R 5′-GATCGATAATACGACTCACTATAGGCCCAATGATCTCACA -3′) that incorporated the T7 RNA polymerase promoter sequence into the PCR amplicon. This riboprobe recognized all three known *Limk2* transcripts. Primers used to amplify *Limk1* were F: 5′- CTACAATTAACCCTCACTAAAGGAAGCGAGTTG -3′ and R: 5′- GATCGATAATACGACTCACTATAGGCACCTGAAGCAGTCTG -3′. The DNA template was used for *in vitro* transcription reaction with 50 μCi of αP33- UTP (Perkin-Elmer). After hybridization at 60°C for 16 hours, sections were treated with RNase and washed in SSC buffer. Slides were dehydrated in a graded ethanol series and exposed to a 75% solution of autoradiographic emulsion type NTB2 (Eastman Kodak Company, Rochester, NY) for 6 to 9 days. Slides were developed using standard protocols [31]. Digital images were acquired with a Hamamatsu ORCA II cooled CCD camera mounted on an Olympus BX60 microscope equipped with dark-field.

**Figure 7:**
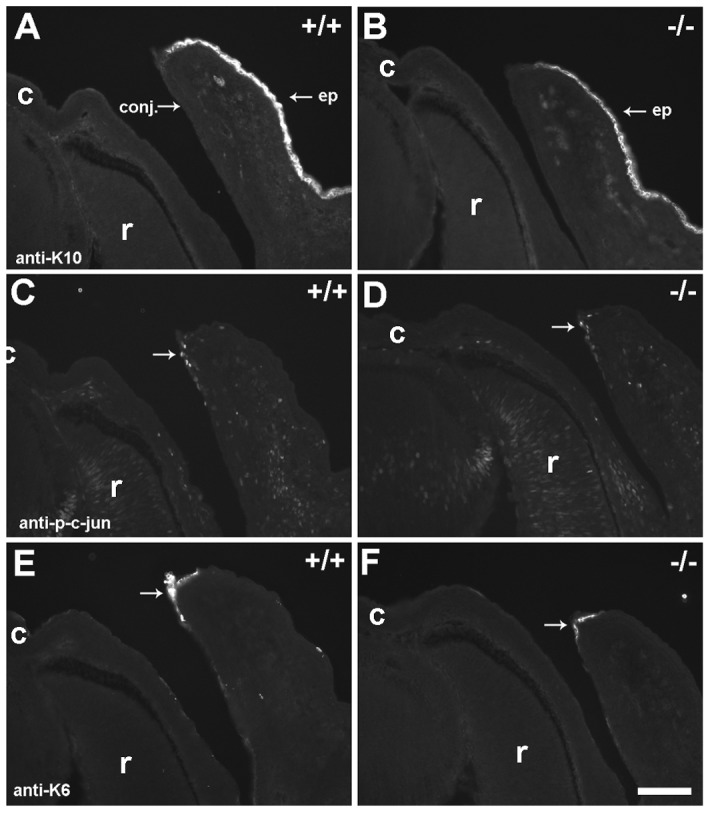
Specification and differentiation of the ocular adnexa at E15.5. Cryosections were stained with various markers of keratinocyte differentiation. For orientation purposes c  =  cornea and r  =  retina in all images. Keratin 10 (K10) is expressed in papebral epidermis (ep) in wild type (A, +/+) and LIMK2-deficient (B, −/−) eyelids. The conjunctiva (conj.) is negative for K10 expression. An antibody recognizing phosphorylated c-Jun labels nuclei in cells (arrows) at the tip of the eyelid in both +/+ (C) and −/− (D) mice. Keratin 6 (K6) is expressed in epithelial cells at the tip of the eyelids in both +/+ (E) and −/− (F) mice, consistent with induction of a migratory phenotype. Scale bar  = 100 μm.

For immunohistochemistry, a rabbit monoclonal antibody (Epitomics, #1795; the immunogen is a synthetic peptide corresponding to residues near the C-terminus of human LIMK2) was used at a 1∶200 dilution in PBS-T (0.01% Triton-X) on formalin-fixed paraffin sections followed by chromogenic detection using diaminobenzidine peroxidase (Vector Laboratories). Filamentous actin (F-actin) was detected in wholemount eyelid preparations from E15.5 specimens using Alexa488-conjugated Phalloidin (Invitrogen, Eugene, OR) diluted 1∶1,000 in 0.1 M PBS. Keratinocyte differentiation was examined using rabbit anti-keratin 6 (PRB-169P; Covance) and rabbit-anti-keratin 10 (PRB-159P; Covance) at 1∶1,000 and 1∶5,000 dilutions in PBS-T, respectively. A rabbit anti-phospho-c-jun-Ser63 antibody (#9261; Cell Signaling) was used at a dilution of 1∶100 in PBS-T. Alexa488 conjugated goat anti-rabbit secondary antibodies were used at 1∶400 in PBS-T. Incubations were from 1 hour to overnight, specimens were washed in PBS and coverslips were applied using Fluoromount G (Electron Microscopy Sciences, Hatfield, PA). Images were acquired using a Hamamatsu ORCA II cooled CCD camera mounted on an Olympus BX60 fluorescence microscope and a Leica confocal microscope equipped with an Argon laser.

**Figure 8:**
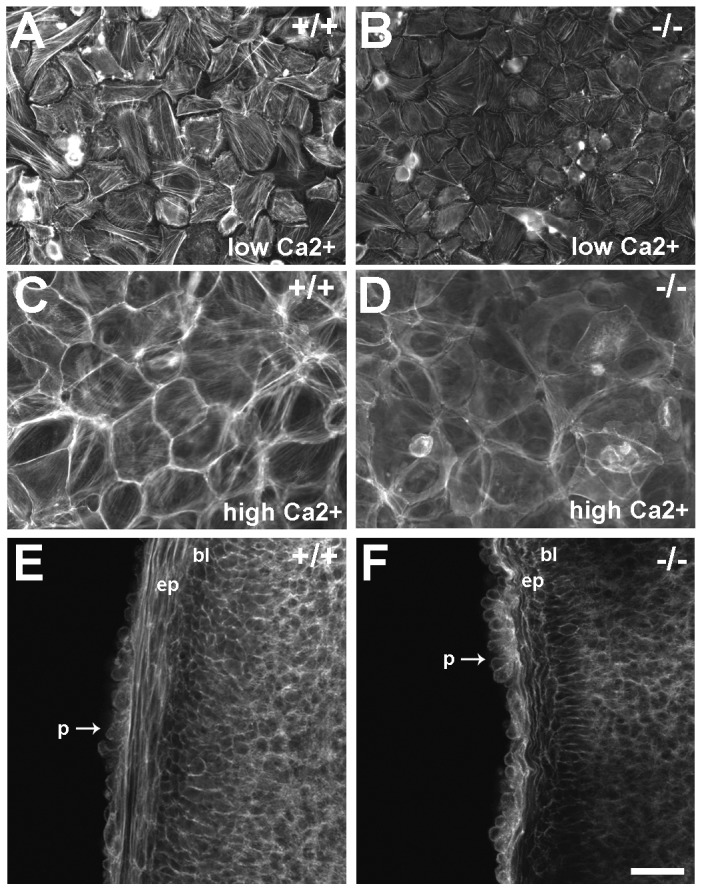
Phalloidin staining of filamentous actin in primary keratinocytes *in vitro* and developing eyelids *in vivo*. Primary keratinocytes were isolated from newborn pups and cultured in either low (60 μM; A, B) or high (1.2 mM; C, D) Ca^2+^ to induce differentiation. (A) Abundant stress fibers are apparent in wild type (+/+) keratinocytes. However, the intensity of phalloidin staining is decreased in LIMK2-deficient keratinocytes (B, −/−). The micrographs in A and B were taken at equal exposures. (C) After stimulation with Ca^2+^ for 24 hours, prominent cortical actin and stress fibers are present in +/+ keratinocytes. (D) Although the overall cell morphology in LIMK2-deficient keratinocytes is comparable to wild type cells, the intensity of cortical phalloidin stain is decreased. (E) Confocal microscopy of wholemount E15.5 ocular adnexa stained with phalloidin reveals intense stress fibers in the +/+ epithelium (ep) adjacent to the basal layer (bl). Cortical actin is also evident in the leading periderm cells (p) in the migrating tip. (F) Although cortical actin is obvious in the −/− periderm cells, stress fibers were not readily apparent in the adjacent epithelium. Scale bar  = 50 μm in A–D and 75 μm in E and F.

For immunoblotting, tissues were lysed in buffer containing: 50 mM Tris, pH 7.5; 250 mM NaCl; 2.5 mM EDTA; 1% IGEPAL CA-630) supplemented with HALT protease and phosphatase inhibitor (Thermo Scientific; 78443). Proteins were resolved on 12% Bis-Tris polyacrylamide gels in SDS running buffer (Invitrogen; WG1403) and transferred to PVDF regular membranes (Invitrogen; LC2005). Membranes were blocked in 5% nonfat milk diluted in Tris-buffered saline plus 0.1% Tween-20 (TBST) and exposed to 1∶1000 rabbit anti-LIMK2 C-terminal (AbCam; EP969Y), or 1∶2000 rat anti-LIMK2 N-terminal monoclonal antibody clone 10E5-19 [32]. Blots were washed and probed with a 1∶5000 donkey anti-rabbit, HRP conjugated secondary antibody (Thermo Scientific; 31458) or 1∶5000 goat anti-rat, HRP conjugated secondary antibody (Southern Biotech; 3050-05). Chemiluminescence was induced using SuperSignal West Pico Chemiluminescent Substrate (Thermo; 34080) and captured on Biomax film (Kodak; 178 8207).

### Keratinocyte culture

Newborn (0–2 days old) mouse pups were obtained from breeding heterozygous LIMK2 knockout mice. Mice were euthanized and a single dorsal incision was made along the length of the body to enable removal of skin. Skin was placed in ice-cold Dulbecco's phosphate buffered saline (DPBS, without calcium and magnesium; Life Technologies) containing 1% antibiotic-antimycotic. Each skin was subsequently rinsed in 70% ethanol for 1 minute, ddH2O for 2 minutes and DPBS with 1% antibiotic-antimycotic for 2 minutes. Skin was then transferred to a 35 mm dish, flattened with dermis side down and incubated in 1 ml of 5 mg/ml dispase solution (Stemcell technologies, Vancouver, Canada) at 4°C overnight. Epidermis was separated from dermis, washed in DPBS and incubated in 2 ml of TrypLE Express (Life Technologies) at 37°C for 30 minutes with periodic agitation. TrypLE was inactivated by dilution with 5 ml of keratinocyte growth medium (KGM: EpiLife medium with 60 µM calcium, 1% Human Keratinocyte Growth Supplement, 1% antibiotic-antimycotic and 1% GlutaMAX, Life Technologies). The cell suspension was filtered through 70 µm cell strainer and centrifuged at 1,000 rpm for 10 minutes at room temperature. Cell pellets were re-suspended in KGM and plated on Collagen I coated chamber slides or plates at 5×10^4^ cells per cm^2^. Cells were re-fed at 24 hours and again every other day. All experiments were carried out at 2–6 days after plating. To induce cell-cell junctions, extracellular calcium levels were increased to 1.2 mM for 24 hours.

Cells were lysed in RIPA buffer (Santa Cruz) containing HALT protease and phosphatase inhibitor cocktail (Thermo Scientific). Cell lysates were centrifuged at 14,000 rpm for 10 min at 4°C. Supernatant was transferred to a new tube and protein concentration was determined by BCA assay (Thermo Scientific). Lysates (approximately 2.1 ug total protein per well) were resolved on 4–12% Bis-Tris polyacrylamide gels (#WG1403; Life Technologies) in 1X, NuPAGE, MES SDS running buffer (#NP0002). Gels were then transferred for 7 minutes to PVDF regular membranes (#IB4010-01) via Life Technologies iBlot dry blotting system. Membranes were rinsed in distilled water then blocked in 5% non-fat dry milk diluted in TBST and exposed to rabbit anti-p-cofilin, (1∶1,000 dilution, Cell Signaling #3313), rabbit anti-cofilin (1∶1,000 dilution, Cell Signaling #3312), or rabbit anti-GAPDH (1∶2000 dilution, Cell Signaling #2118). Blots were washed and probed with an anti-rabbit, HRP conjugated secondary antibody from GE Healthcare (1∶20,000 dilution, NA934V). Chemiluminescence was induced using ECL Prime kit from GE Healthcare (RPN2232) and captured on the VersaDoc imaging system (BioRad, UK).

## Results

### Generation and Molecular Characterization of *Limk2*-Deficient Mice

To study the function of the *Limk2* gene, we chose to disrupt expression of all known *Limk2* transcripts using gene trapping. To generate the gene trap allele, we searched the OmniBank sequence database [26], [27] by BLAST using both cDNA and genomic sequence from the *Limk2* gene interval and identified several mouse embryonic stem (ES) cell clones predicted to contain gene trap mutations within the *Limk2* gene. ES cell clone OST80053 (accession CG518476) was selected for further characterization based on the sequence similarity of its 3′ RACE (rapid amplification of cDNA ends) tag with exons that code for the kinase domain. Inverse genomic PCR was used to amplify the vector insertion site from this clone and localize the insertion mutation to intron 12 of the *Limk2* gene (Fig. 1A). The mutation was determined to occur downstream of the initiation codons for all reported *Limk2* transcripts (Fig. 1A), suggesting that all isoforms should be disrupted. Interbreeding of F1 pure hybrid *Limk2* +/− mice gave rise to F2 progeny, that are randomly segregating 129S5SvEvBrd and C57BL/6-Tyr*^c-Brd^* alleles, in the predicted Mendelian ratio (75+/+, 158 +/− and 75−/− mice). To confirm the disruption of *Limk2* transcription *in vivo*, we performed reverse transcription-PCR (RT-PCR) using primers complementary to exonic sequences flanking the vector insertion site. No endogenous *Limk2* transcript was detected in several *Limk2* (−/−) tissues (Fig. 1B). Immunoblotting analysis with a monoclonal antibody recognizing residues 145–260, which include the PDZ domain, revealed LIMK2 in several tissues such as spleen, brain, lung and retina in wild type mice (+/+). No detectable LIMK2 was observed in these tissues isolated from homozygous *Limk2* (−/−) mutant mice (Fig. 1C). Primary keratinocytes were isolated from neonates to further explore the biochemical consequences of LIMK2 knockout. Immunoblots of primary keratinocytes were probed with antibodies that recognize either total cofilin, a biochemical substrate of LIMK2, or phosphorylated cofilin (Ser-3). In LIMK2-deficient keratinocytes, the ratio of phospho-cofilin to total cofilin was decreased approximately 65% compared to wild type controls (Fig. 1D). This result was statistically significant (P<0.001; *n* = 4 per genotype) and demonstrated the loss of LIMK2 catalytic function in the knockout line. The residual phosphorylation of cofilin could be due to the fact that other kinases are known to phosphorylate this ABP [17].

### Mice Deficient in LIMK2 Exhibit Ocular Phenotypes

Mice deficient in *Limk2* exhibited normal behavior, thrived to adulthood and were fertile. Upon close examination of the eye, *Limk2* knockouts were observed to have shallow anterior chambers and corneal neovascularization. The anterior segment phenotypes in the *Limk2* knockout closely resembled phenotypes previously reported in *Rock2*-deficient mice [33]. To explore the potential conservation of the ROCK-LIMK pathway in ocular biology, we examined eyelid development in *Limk2*-deficient mice.

### Mice Deficient in LIMK2 Exhibit an Eyelid Open at Birth (EOB) Phenotype

*Limk2*-deficient homozygous mice (−/−) exhibited eyelids that were open at birth (arrow Fig. 2B) compared to fused eyelids observed in wild type mice (+/+; Fig. 2A). Histopathology of postnatal day 1.5 (PD 1.5) wild type eyes revealed a fusion zone (arrow Fig. 2C) between the upper and lower eyelid epidermis. Superficial to this fusion zone, the epidermis contained the eosinophilic stratum corneum. The upper and lower eyelids remained separated in the *Limk2* knockout mice (Fig. 2D). Histological changes observed in these young mice included keratinization, inflammation and edema centered on exposed areas of the cornea. In some areas, the corneal surface was covered with a thick serocellular crust.

Eyelids in both wild type and heterozygous *Limk2* mice were completely fused at E18.5, prior to birth (Fig. 3A, C). The zone of upper and lower eyelid fusion was clearly recognizable and stratum corneum was observed in the palpebral epidermis (arrowheads in Fig. 3C). The inner surface of the eyelid, which corresponds to the palpebral conjunctiva, exhibited non-keratinized epithelium. Eyelids in *Limk2*-deficient mice were open at E18.5 (Fig. 3B, D). Otherwise, the ocular histology was comparable to that in controls. Specification of the palpebrae was normal, with a stratum corneum (arrowhead in Fig. 3D) on the epidermis and a non-keratinized epithelium on the conjunctival aspect. There were no signs of inflammation in the *Limk2*-deficient eyes prior to birth. Therefore, the most likely explanation for the observed histopathology in postnatal *Limk2*-deficient mice is exposure of the ocular surface to environmental irritants during and after birth.

### The EOB Phenotype Arises From a Failure in Eyelid Fusion

Eyelid closure occurs in all mammalian species, albeit at different gestational periods. In mice, eyelid closure occurs by embryonic day 16 (E16) and eyelids remain closed until the second postnatal week [3]. The EOB phenotype in *Limk2*-deficient mice could arise from premature eyelid opening or a failure in eyelid closure. To distinguish between these two possibilities, timed-matings were performed to examine embryos ranging in age from E14.5 to E16.5.

Critical events in eyelid closure occur between E14.5 and E16.5 [3]. The eyelid contains two major anatomical divisions at E14.5, the superficial epithelium (future epidermis) and the underlying mesenchyme that will develop into the dermis. At this age in both control and *Limk2*-deficient eyelids, peridermal cells lined the external surface of the epithelium and exhibited the squamous cell morphology. The underlying epithelium (ep) contained several rows of cell nuclei at E14.5 (Fig. 4A, B). Specialized periderm cells (arrows in Fig. 4) emerged from the tip of the eyelid epithelium around E14.5 to E15. These cells were round in appearance and developed from the periderm of the eyelid epithelium in control mice (Fig. 4A) and mice lacking *Limk2* (Fig. 4B). There was no discernible difference in eyelid or cornea anatomy in *Limk2*-deficient mice compared to either wild type or heterozygous controls at E14.5.

Approximately 24 hours later, at E15.5, the EOB phenotype became apparent in homozygous *Limk2* mice (Fig. 4C, D). In wild type or heterozygous mice, the cluster of round peridermal cells, first observed at the tip of the eyelid epithelium on E14.5, became a leading edge of cells that extended over the cornea (p in Fig. 4C). Periderm-like cells, exhibiting squamous cell morphology, formed the cortex of the extension behind the population of round cells leading the front. Epithelial cells (ep in Fig. 4C) were observed within eyelid extensions of both the upper and lower lids. The morphology of these epithelial cells contrasted with the basal layer (bl in Fig. 4C, D) of epithelium adjacent to the mesenchyme. In homozygous *Limk2* mice, the population of round cells remained closely associated with the tip of the eyelid epithelium (Fig. 4D). No discernible difference in eyelid closure or anatomy was observed between wild type and heterozygous mice, suggesting that a single copy of a functional *Limk2* allele provided sufficient biochemical activity to drive eyelid epithelial cell extension. The EOB phenotype was observed in 100% of the *Limk2* −/− mice (*n* = 21) studied between E15.5 and postnatal day 9.5 (PD 9.5).

### Epithelial Cells in the Developing Eyelid Sheet Express LIMK2

*In situ* hybridization and immunohistochemistry were performed to investigate cell populations that express *Limk2* during the critical period of eyelid closure. Isotopic *in sit*u hybridization revealed *Limk2* expression in wild type (+/+) epithelium (ep) in both the upper and lower eyelids (two different cases shown in Fig. 5A, D). Epithelium in the cornea (c) and neurons in the ganglion cell layer (gcl) of the neural retina also express *Limk2*. Significantly, the emerging epithelial cells at the eyelid tips exhibited higher levels of *Limk2* compared with adjacent epithelium (arrows in Fig. 5A, D). This was also observed in *Limk2* heterozygous mice (+/−). In contrast, hybridization signals were greatly diminished in homozygous (−/−) *Limk2*-deficient mice (Fig. 5C). *In situ* hybridization using a riboprobe specific for *Limk1* revealed expression in neurons located in the gcl and no detectable expression in the eyelid epithelium (Fig. 5E). Hybridization on an adjacent section with the *Limk1* sense probe was negative (Fig. 5F).

Immunohistochemistry with an antibody recognizing LIMK2 was performed on paraffin sections obtained from E15.5 embryos. Epithelial sheets migrating from both upper and lower eyelids expressed high levels of LIMK2 (Fig. 6A). The relatively high levels of LIMK2 signal in the migrating epithelial front compared with adjacent epithelium is consistent with the results obtained with the *Limk2* riboprobe. No LIMK2 immunoreactivity was observed with homozygous (−/−) *Limk2*-deficient mice (Fig. 6B), indicating the specificity of this reagent. In control mice, higher magnification revealed LIMK2 staining in migrating epithelial cells (ep) and rounded periderm (p) cells in the leading edge (Fig. 6C). LIMK2 immunoreactivity was also observed in corneal epithelium and periderm cells. The mesenchymal (m) and corneal stroma expressed either very low levels or undetectable levels of LIMK2. Rounded periderm cells were present in the *Limk2*-deficient mice at margins of the upper and lower eyelids, but no detectable LIMK2 was observed in these cells or corneal periderm and epithelial cells (Fig. 6D).

### Palpebrae Specification and Expression of Markers Indicative of Keratinocyte Migration are Normal in LIMK2 Deficient Eyelids

Genetic studies have identified several signal transduction pathways important for eyelid closure in mice. Broadly speaking, these can be characterized into two major signaling cascades initiated either by the TGFβ superfamily of ligands (including inhibins and bone morphogenetic proteins; BMPs), or EGFR ligands (such as EGF and TGFα). Knockout of SMAD4 yields the prototypical phenotypes observed in eyelids deficient in canonical BMP signaling [34]. In these knockouts, the conjunctival epithelium exhibited features of abnormal specification, including keratinization, ectopic hair follicles and expression of keratin 10, a marker normally restricted to the palpebral epidermis. No evidence of abnormal localization of keratin 10 was found in eyelids of developing LIMK2-deficient mice compared to controls (Fig. 7A, B). During eyelid closure, deficiency in TGFβ-signaling, arising through either SMAD-dependent or MAP3K1/JNK-dependent biochemical pathways, is associated with decreased levels of phosphorylated c-Jun in nuclei of migrating periderm cells and keratinocytes *in vivo* and primary keratinocytes *in vitro* [34]–[36]. In contrast, phosphorylated c-Jun was observed in nuclei of cells emerging from the eyelid tip in LIMK2-deficient embryos and their wild type controls (Fig. 7C, D). Deficiency in JNK or c-Jun reduces EGF expression and EGFR phosphorylation in developing eyelid sheets and keratinocytes *in vitro* [35], [37]. EGFR ligands are known to induce expression of keratin 6, a marker of activated keratinocytes that are migratory in nature and that respond to growth factors [38]. However, keratin 6 expression in the tip of the emerging eyelid epithelium was similar in LIMK2-deficient and wild type mice (Fig. 7 E, F). Taken together, these results suggest that the classical ligand-receptor pathways associated with EOB phenotypes remain intact in LIMK2-deficient mice and that keratinocytes in the eyelid tip exhibit a pro-migratory phenotype.

LIMK2 deficiency in keratinocytes resulted in deceased levels of p-cofilin (Fig. 1D), which might be expected to alter actin filament structure. Phalloidin staining was used on primary keratinocytes and embryonic eyelid samples to examine filamentous actin. Primary keratinocytes isolated from wild type (+/+) neonates exhibited intense stress fiber staining in low calcium (Fig. 8A). Although stress fibers were present in LIMK2-deficient keratinocytes, the intensity of phalloidin stain was reproducibly decreased compared to controls (Fig. 8B). Stimulation of wild type primary keratinocytes with high calcium induced a morphological change in keratinocytes characterized by prominent cortical actin (Fig. 8C). In LIMK2-deficient keratinocytes stimulated with high calcium, the intensity of phalloidin staining remained decreased and cortical actin was not observed (Fig. 8D). *In vivo*, phalloidin staining in wild type embryos at E15.5 revealed intense staining of stress fibers in the epithelial sheet emerging from the eyelid tip (Fig. 8E). The intensity of stress fiber staining in the epithelium was consistently reduced in LIMK2-deficient embryos (Fig. 8F).

## Discussion

Ocular phenotypes in the LIMK2 knockout described in this work closely resemble those in ROCK knockouts [12], [13], [33]. In each knockout model, keratinocytes emerging from the tip of the eyelid fail to nucleate filamentous actin and subsequently do not migrate. These cells normally express high levels of LIMK2 and ROCKs suggesting that the migration defect is cell intrinsic. LIMK2-deficient keratinocytes also exhibit decreased levels of phosphorylated (i.e. inactive) cofilin and decreased stress fibers, most likely due to increased actin severing by cofilin and/or another ABP. Previous work has shown that ROCKs phosphorylate several substrates that modulate actin dynamics, but the critical downstream targets of ROCK signaling during eyelid closure remained unknown. The EOB phenotype in the LIMK2 genetic model identifies a ROCK biochemical target that modulates actin dynamics during keratinocyte migration important for eyelid closure.

A knockout of *Limk2* was previously published, but there was no mention of an ocular phenotype and attention was placed on phenotypes observed in the testes [39]. Testicular weights were decreased (≈15%) in the knockout and increased apoptosis was observed in seminiferous tubules. Despite these findings, male *Limk2* knockout mice were reported as fertile [39]. The *Limk2* knockout described here is on a different genetic background, exhibits a profound EOB phenotype and testicular phenotypes (weight and histology) were not observed in comparable aged mice. Penetrance of EOB phenotypes is known to be influenced by genetic background [8], [10], which is the most likely explanation for the phenotypic discrepancy observed in these two *Limk2* knockout lines.

Genetic studies have identified several pathways that are necessary for actin polymerization and eyelid closure [6]–[10]. The EGFR ligands, HB-EGF and TGFα activate ERK and promote F-actin accumulation in migrating keratinocytes. TGF-β/activin stimulates the MAP3K1-JNK pathway, which induces actin polymerization and phosphorylation of c-Jun in the leading migratory cells. Levels of phosphorylated-c-Jun are dramatically decreased in the MAP3K1 knockout eyelid epithelial sheet [36]. Conditional ablation of c-jun in keratinocytes results in an EOB phenotype associated with reduced levels of EGFR and HB-EGF in the developing eyelid epithelial sheet. This observation suggests that c-jun serves as a positive feedback signal to promote the EGFR pathway in migrating keratinocytes. However, JNK activation and subsequent c-Jun phosphorylation are not altered in ROCK1 knockout keratinocytes [12] or the LIMK2 knockout characterized here. This result implies that MAPK, ERK and JNK pathways are unable to compensate for loss of the ROCK-LIMK2 pathway and vice versa. Both pathways ultimately converge on serum response factor (SRF) to independently induce gene expression through recruitment of different SRF transcriptional cofactors [40], [41]. Conditional ablation of *Srf* in keratinocytes leads to an EOB phenotype [42] and LIMKs have previously been identified as direct activators of SRF-mediated transcription in certain cell types [43], [44]. We are currently investigating the possibility that SRF coordinates input from MAPK and ROCK signaling pathways to control expression of genes affecting keratinocytes migration.

## References

[1] Martin P, Parkhurst SM (2004) Parallels between tissue repair and embryo morphogenesis. Development 131: 3021–3034. 15197160 10.1242/dev.01253

[2] Vaezi A, Bauer C, Vasioukhin V, Fuchs E (2002) Actin cable dynamics and Rho/Rock orchestrate a polarized cytoskeletal architecture in the early steps of assembling a stratified epithelium. Dev Cell 3: 367–381. 12361600 10.1016/s1534-5807(02)00259-9

[3] Findlater GS, McDougall RD, Kaufman MH (1993) Eyelid development, fusion and subsequent reopening in the mouse. J Anat 183 ( Pt 1): 121–129. PMC1259860 8270467

[4] Harris MJ, Juriloff DM (1986) Eyelid development and fusion induced by cortisone treatment in mutant, lidgap-Miller, foetal mice. A scanning electron microscope study. J Embryol Exp Morphol 91: 1–18. 3711778

[5] Maconnachie E (1979) A study of digit fusion in the mouse embryo. J Embryol Exp Morphol 49: 259–276. 448272

[6] Miettinen PJ, Berger JE, Meneses J, Phung Y, Pedersen RA, et al (1995) Epithelial immaturity and multiorgan failure in mice lacking epidermal growth factor receptor. Nature 376: 337–341. 7630400 10.1038/376337a0

[7] Sibilia M, Wagner EF (1995) Strain-dependent epithelial defects in mice lacking the EGF receptor. Science 269: 234–238. 7618085 10.1126/science.7618085

[8] Threadgill DW, Dlugosz AA, Hansen LA, Tennenbaum T, Lichti U, et al (1995) Targeted disruption of mouse EGF receptor: effect of genetic background on mutant phenotype. Science 269: 230–234. 7618084 10.1126/science.7618084

[9] Luetteke NC, Qiu TH, Peiffer RL, Oliver P, Smithies O, et al (1993) TGF alpha deficiency results in hair follicle and eye abnormalities in targeted and waved-1 mice. Cell 73: 263–278. 8477445 10.1016/0092-8674(93)90228-i

[10] Mine N, Iwamoto R, Mekada E (2005) HB-EGF promotes epithelial cell migration in eyelid development. Development 132: 4317–4326. 16141218 10.1242/dev.02030

[11] Hall A (2005) Rho GTPases and the control of cell behaviour. Biochem Soc Trans 33: 891–895. 16246005 10.1042/BST20050891

[12] Shimizu Y, Thumkeo D, Keel J, Ishizaki T, Oshima H, et al (2005) ROCK-I regulates closure of the eyelids and ventral body wall by inducing assembly of actomyosin bundles. J Cell Biol 168: 941–953. 15753128 10.1083/jcb.200411179 PMC2171774

[13] Thumkeo D, Shimizu Y, Sakamoto S, Yamada S, Narumiya S (2005) ROCK-I and ROCK-II cooperatively regulate closure of eyelid and ventral body wall in mouse embryo. Genes Cells 10: 825–834. 16098146 10.1111/j.1365-2443.2005.00882.x

[14] Riento K, Ridley AJ (2003) Rocks: multifunctional kinases in cell behaviour. Nat Rev Mol Cell Biol 4: 446–456. 12778124 10.1038/nrm1128

[15] Amano T, Tanabe K, Eto T, Narumiya S, Mizuno K (2001) LIM-kinase 2 induces formation of stress fibres, focal adhesions and membrane blebs, dependent on its activation by Rho-associated kinase-catalysed phosphorylation at threonine-505. Biochem J 354: 149–159. 11171090 10.1042/0264-6021:3540149 PMC1221639

[16] Bernard O (2007) Lim kinases, regulators of actin dynamics. Int J Biochem Cell Biol 39: 1071–1076. 17188549 10.1016/j.biocel.2006.11.011

[17] Scott RW, Olson MF (2007) LIM kinases: function, regulation and association with human disease. J Mol Med 85: 555–568. 17294230 10.1007/s00109-007-0165-6

[18] Yoshioka K, Foletta V, Bernard O, Itoh K (2003) A role for LIM kinase in cancer invasion. Proc Natl Acad Sci U S A 100: 7247–7252. 12777619 10.1073/pnas.1232344100 PMC165861

[19] Manetti F (2011) LIM kinases are attractive targets with many macromolecular partners and only a few small molecule regulators. Med Res Rev. 10.1002/med.20230 22886629

[20] Arber S, Barbayannis FA, Hanser H, Schneider C, Stanyon CA, et al (1998) Regulation of actin dynamics through phosphorylation of cofilin by LIM-kinase. Nature 393: 805–809. 9655397 10.1038/31729

[21] Maekawa M, Ishizaki T, Boku S, Watanabe N, Fujita A, et al (1999) Signaling from Rho to the actin cytoskeleton through protein kinases ROCK and LIM-kinase. Science 285: 895–898. 10436159 10.1126/science.285.5429.895

[22] Sumi T, Matsumoto K, Takai Y, Nakamura T (1999) Cofilin phosphorylation and actin cytoskeletal dynamics regulated by rho- and Cdc42-activated LIM-kinase 2. J Cell Biol 147: 1519–1532. 10613909 10.1083/jcb.147.7.1519 PMC2174243

[23] Yang N, Higuchi O, Ohashi K, Nagata K, Wada A, et al (1998) Cofilin phosphorylation by LIM-kinase 1 and its role in Rac-mediated actin reorganization. Nature 393: 809–812. 9655398 10.1038/31735

[24] Moon A, Drubin DG (1995) The ADF/cofilin proteins: stimulus-responsive modulators of actin dynamics. Mol Biol Cell 6: 1423–1431. 8589446 10.1091/mbc.6.11.1423 PMC301301

[25] Scott RW, Hooper S, Crighton D, Li A, Konig I, et al (2010) LIM kinases are required for invasive path generation by tumor and tumor-associated stromal cells. J Cell Biol 191: 169–185. 20876278 10.1083/jcb.201002041 PMC2953444

[26] Zambrowicz BP, Friedrich GA, Buxton EC, Lilleberg SL, Person C, et al (1998) Disruption and sequence identification of 2,000 genes in mouse embryonic stem cells. Nature 392: 608–611. 9560157 10.1038/33423

[27] Zambrowicz BP, Abuin A, Ramirez-Solis R, Richter LJ, Piggott J, et al (2003) Wnk1 kinase deficiency lowers blood pressure in mice: A gene-trap screen to identify potential targets for therapeutic intervention. Proc Natl Acad Sci U S A 10: 10. 10.1073/pnas.2336103100 PMC283554 14610273

[28] Joyner AL (2000) Gene Targeting: A Practical Approach. Oxford, England: Oxford University Press.

[29] Hansen GM, Markesich DC, Burnett MB, Zhu Q, Dionne KM, et al (2008) Large-scale gene trapping in C57BL/6N mouse embryonic stem cells. Genome Res 18: 1670–1679. 18799693 10.1101/gr.078352.108 PMC2556270

[30] Rice DS, Sheldon M, D'Arcangelo G, Nakajima K, Goldowitz D, et al (1998) Disabled-1 acts downstream of Reelin in a signaling pathway that controls laminar organization in the mammalian brain. Development 125: 3719–3729. 9716537 10.1242/dev.125.18.3719

[31] Simmons DM, Arriza JL, Swanson LW (1989) A complete protocol for *in situ* hybridization of messenger RNAs in brain and other tissues with radiolabeled single-stranded RNA probes. J Histotech 12: 169–181.

[32] Acevedo K, Moussi N, Li R, Soo P, Bernard O (2006) LIM kinase 2 is widely expressed in all tissues. J Histochem Cytochem 54: 487–501. 16399995 10.1369/jhc.5C6813.2006

[33] Whitlock NA, Harrison B, Mixon T, Yu XQ, Wilson A, et al (2009) Decreased intraocular pressure in mice following either pharmacological or genetic inhibition of ROCK. J Ocul Pharmacol Ther 25: 187–194. 19456252 10.1089/jop.2008.0142

[34] Huang J, Dattilo LK, Rajagopal R, Liu Y, Kaartinen V, et al (2009) FGF-regulated BMP signaling is required for eyelid closure and to specify conjunctival epithelial cell fate. Development 136: 1741–1750. 19369394 10.1242/dev.034082 PMC2673764

[35] Weston CR, Wong A, Hall JP, Goad ME, Flavell RA, et al (2004) The c-Jun NH2-terminal kinase is essential for epidermal growth factor expression during epidermal morphogenesis. Proc Natl Acad Sci U S A 101: 14114–14119. 15375216 10.1073/pnas.0406061101 PMC521127

[36] Zhang L, Wang W, Hayashi Y, Jester JV, Birk DE, et al (2003) A role for MEK kinase 1 in TGF-beta/activin-induced epithelium movement and embryonic eyelid closure. EMBO J 22: 4443–4454. 12941696 10.1093/emboj/cdg440 PMC202382

[37] Zenz R, Scheuch H, Martin P, Frank C, Eferl R, et al (2003) c-Jun regulates eyelid closure and skin tumor development through EGFR signaling. Dev Cell 4: 879–889. 12791272 10.1016/s1534-5807(03)00161-8

[38] Jiang CK, Magnaldo T, Ohtsuki M, Freedberg IM, Bernerd F, et al (1993) Epidermal growth factor and transforming growth factor alpha specifically induce the activation- and hyperproliferation-associated keratins 6 and 16. Proc Natl Acad Sci U S A 90: 6786–6790. 7688128 10.1073/pnas.90.14.6786 PMC47017

[39] Takahashi H, Koshimizu U, Miyazaki J, Nakamura T (2002) Impaired spermatogenic ability of testicular germ cells in mice deficient in the LIM-kinase 2 gene. Dev Biol 241: 259–272. 11784110 10.1006/dbio.2001.0512

[40] Gineitis D, Treisman R (2001) Differential usage of signal transduction pathways defines two types of serum response factor target gene. J Biol Chem 276: 24531–24539. 11342553 10.1074/jbc.M102678200

[41] Olson EN, Nordheim A (2010) Linking actin dynamics and gene transcription to drive cellular motile functions. Nat Rev Mol Cell Biol 11: 353–365. 20414257 10.1038/nrm2890 PMC3073350

[42] Verdoni AM, Ikeda S, Ikeda A (2010) Serum response factor is essential for the proper development of skin epithelium. Mamm Genome 21: 64–76. 20047077 10.1007/s00335-009-9245-y PMC2872102

[43] Sotiropoulos A, Gineitis D, Copeland J, Treisman R (1999) Signal-regulated activation of serum response factor is mediated by changes in actin dynamics. Cell 98: 159–169. 10428028 10.1016/s0092-8674(00)81011-9

[44] Geneste O, Copeland JW, Treisman R (2002) LIM kinase and Diaphanous cooperate to regulate serum response factor and actin dynamics. J Cell Biol 157: 831–838. 12034774 10.1083/jcb.200203126 PMC2173419

[45] Ikebe C, Ohashi K, Mizuno K (1998) Identification of testis-specific (Limk2t) and brain-specific (Limk2c) isoforms of mouse LIM-kinase 2 gene transcripts. Biochem Biophys Res Commun 246: 307–312. 9610354 10.1006/bbrc.1998.8609

[46] Takahashi H, Koshimizu U, Nakamura T (1998) A novel transcript encoding truncated LIM kinase 2 is specifically expressed in male germ cells undergoing meiosis. Biochem Biophys Res Commun 249: 138–145. 9705845 10.1006/bbrc.1998.9094

